# Residual Impact of Concurrent, Resistance, and High-Intensity Interval Training on Fasting Measures of Glucose Metabolism in Women With Insulin Resistance

**DOI:** 10.3389/fphys.2021.760206

**Published:** 2021-11-11

**Authors:** Cristian Alvarez, Emmanuel Gomes Ciolac, Guilherme Veiga Guimarães, David C Andrade, Manuel Vasquez-Muñoz, Matías Monsalves-Álvarez, Pedro Delgado-Floody, Alicia M. Alonso-Martínez, Mikel Izquierdo

**Affiliations:** ^1^Quality of Life and Wellness Research Group, Department of Health, Universidad de Los Lagos, Osorno, Chile; ^2^Exercise and Chronic Disease Research Laboratory, Department of Physical Education, School of Sciences, São Paulo State University (UNESP), São Paulo, Brazil; ^3^Heart Institute, School of Medicine, University of São Paulo, São Paulo, Brazil; ^4^Centro de Investigación en Fisiología y Medicina de Altura (FiMedAlt), Biomedical Department, Facultad de Ciencias de la Salud, Universidad de Antofagasta, Antofagasta, Chile; ^5^Clinica Santa Maria, Santiago, Chile; ^6^Instituto de Ciencias de La Salud, Universidad de O’higgins, Rancagua, Chile; ^7^Human Performance Laboratory, Motion Health and Performance Center, Lo Barnechea, Chile; ^8^Department of Physical Education, Sports and Recreation, Universidad de La Frontera, Temuco, Chile; ^9^Navarrabiomed, Hospital Universitario de Navarra (HUN), Universidad Pública de Navarra (UPNA), IdiSNA, Pamplona, Spain; ^10^CIBER of Frailty and Healthy Aging (CIBERFES), Instituto de Salud Carlos III, Madrid, Spain

**Keywords:** insulin resistance, resistance training, high-intensity interval training, concurrent training, metabolism

## Abstract

We sought to assess the residual effects (post 72-h training cessation) on fasting plasma glucose (FPG) and fasting insulin (FI) after 12-weeks of high-intensity interval training (HIIT), resistance training (RT), or concurrent training (CT) in women with insulin resistance (IR). We also aimed to determine the training-induced, post-training residual impact of CT. A total of adult 45 women (age 38.5±9.2years) were included in the final analysis and were assigned to a control (CG; *n*=13, BMI 28.3±3.6kg/m^2^), HIIT [*n*=14, BMI 28.6±3.6kg/m^2^, three sessions/wk., 80–100% of the maximum heart rate (HR_max_)], RT [*n*=8, BMI 29.4±5.5kg/m^2^, two sessions/wk., 8–10 points of the modified Borg, corresponding to 20 to 50% range of one maximum repetition test (1RM)], or CT group (*n*=10, BMI 29.1±3.0kg/m^2^, three sessions/wk., 80–100% of HR_max_, and 8–10 Borg, or 20 to 50% range of 1RM, to each HIIT and RT compounds), with the latter including both HIIT and RT regimens. Training interventions lasted 12-weeks. The main outcomes were FPG and FI measured at pre- and 24-h and 72-h post-training (FPG_24h_, FI_24h_, and FPG_72h_, FI_72h_, respectively). Secondary endpoints were body composition/anthropometry and the adiposity markers waist circumference (WC) and tricípital skinfold (T_SF_). The residual effects 72-h post-training [delta (∆)] were significantly poorer (all *p*<0.01) in the CT group (∆FPG_72h_+6.6mg/dl, *η*^2^: 0.76) than in the HIIT (∆FPG_72h_+1.2mg/dl, *η*^2^: 0.07) and RT (∆FPG_72h_+1.0mg/dl, *η*^2^: 0.05) groups. These findings reveal that HIIT reduces FPG and RT reduces FI 24-h post-training; both exercise interventions alone have remarkably better residual effects on FPG and FI (post-72h) than CT in women with insulin resistance.

## Introduction

Insulin resistance (IR) is the inability of the insulin hormone to facilitate glucose uptake from peripheral tissues to meet metabolic demands (Abdul-Ghani and DeFronzo, 2010). IR precedes type 2 diabetes mellitus (T2DM; Abdul-Ghani and DeFronzo, 2010) and is a hallmark of the disease. In 2017, the overall public spending on T2DM reached $ 237 billion in the United States alone (Wang et al., 2017). Among the different available therapies to treat T2DM and its complications, exercise training is a unique, non-pharmacological intervention that improves several physical inactivity-related metabolic disorders including IR (Jenkins and Hagberg, 2011; Slentz et al., 2011; Matos et al., 2018) and is a therapy for T2DM (Little et al., 2018). The major beneficial metabolic effects of exercise training are associated with structural adaptations (i.e., changes to adipose, skeletal muscle, bone tissue, and vessels), and with residual effects (hours or days post-exercise) in at-risk populations (Andersen and Høstmark, 2007; Short et al., 2012; Burke et al., 2017).

It is well known that some training regimens can promote improvements in several cardiometabolic markers (American Diabetes Association, 2017). High-intensity interval training (HIIT) and resistance training (RT) have been recommended for people with poor glucose control (Hayes et al., 2009; Colberg et al., 2016; Colberg, 2017). HIIT promotes greater cardiorespiratory fitness (CRF) and metabolic benefits in populations IR (Jelleyman et al., 2015) and T2DM (Little et al., 2011). Additionally, RT also promotes similar glucose control improvements to HIIT (Ross et al., 2021), by increasing skeletal muscle (Brooks et al., 2007) and bone mass (Wood and O’Neill, 2012) in patients with T2DM (Dunstan et al., 2002; Ross et al., 2021). The combination of moderate-intensity continuous training (MICT) plus RT, or also as HIIT plus RT (Da Silva et al., 2020), resulting in a training regimen known as concurrent training (CT), has also relevant evidence in favor of IR patients (Timmons et al., 2018; Álvarez et al., 2019). Indeed, we recently reported beneficial cardiometabolic outcomes in women with hyperglycemia after a 20-week CT intervention, including −4mg/dl decreases in fasting plasma glucose (FPG), and other physiological adaptations in body composition (−4cm waist circumference, +400*g* increases in lean mass), cardiovascular system (−6/−3mmHg reduction in systolic/diastolic blood pressure), and plasma lipoproteins (−11mg/dl LDL-cholesterol reduction) and greater increases in endurance performance (+56m in the 6-min walking test; Álvarez et al., 2019). While numerous studies have reported improved cardiometabolic health after short- or long-term exercise training with HIIT, RT, or CT, these exercise adaptations are typically short-lived and are reversed after training cessation (detraining), as is the case for metabolic outcomes (Bajpeyi et al., 2009; Del Vecchio et al., 2020). At the level of physiological adaptations from HIIT, RT, and CT, the results are scarce at level of post-exercise cessation, and few studies, and few studies have monitored the residual effects on glucose control markers in IR cohorts (Short et al., 2012).

In the case of CT, some studies have reported “interference effects” when performing HIIT plus RT in the same exercise routine (Wilson et al., 2012; Vechin et al., 2021), while others have found no detrimental interactions in health-related and performance outcomes (Lundberg et al., 2013; Villareal et al., 2017). Specifically, the “interference effect” has been described when MICT, or RT alone promotes a specific molecular profile that generates mitochondrial biogenesis and thus increases fatigue tolerance or hypertrophy, and strength at the skeletal muscle level, but when both regimens are applied concurrently (in the same exercise session), other more specific training adaptations can occur, and endurance training can attenuate the muscle hypertrophy and strength gains (Coffey and Hawley, 2017). Some of the discrepancies in the literature likely arise because of the order of the sessions of CT [i.e., RT+HIIT or HIIT+RT (Bagheri et al., 2020)], or in the comparisons of CT vs. RT or HIIT alone, in which no differences were reported in strength performance increases, but minor cardiorespiratory adaptations are evident when comparing CT vs. MICT or RT alone (Glowacki et al., 2004). In addition, while HIIT has a strong capacity for improving CRF, and marked evidence for reducing adiposity impacting overall adiposity markers, by contrast, RT has a high capacity for increasing skeletal muscle mass and other tissues as bone.

It has been proposed that the beneficial adaptations to exercise training are not related to the training itself, where most of these are linked to the recovery period, or after exercise cessation, which is known as “residual effect.” For example, most events in which glucose control is improved by exercise training (i.e., FPG decreases) are after exercise cessation, major body fat decreases operate similarly after HIIT, or the greater skeletal muscle mass increases are resulting from the sum of physiological events during recovery time. Thus, particularly, the residual effect is the post-exercise cessation time in which the beneficial exercise effects are extended without exercise participation until these physiological adaptations are lost, but at the same time other adaptations are assembled at different systems (tissues, cardiovascular, metabolic among others).

Based on previous reports about some detrimental adaptations to increase strength from CT of endurance plus RT exercise in which RT alone promote more strength increases (Bajpeyi et al., 2009), considering the relevance of increasing skeletal muscle mass for glucose control in IR and T2DM patients (Dunstan et al., 2002; Brooks et al., 2007; Ross et al., 2021), we hypothesized that both HIIT or RT alone, but not CT, would have long-term residual effects on FPG and FI in women with IR. Thus, the present study aimed to assess the residual effects (post-72-h training cessation) on FPG and FI after 12-weeks of HIIT, RT, or CT in adult women with IR.

## Materials and Methods

We studied physically inactive adult women with insulin resistance but with no diagnosis of T2DM. The study was performed in accordance with the ethical standards established by the Declaration of Helsinki (2013) and was approved by the local ethical committee of the Family Healthcare Center TRV of Los Lagos, Chile (no. 0204015). The study was not registered in a database. All participants signed a written informed consent.

Eligibility criteria included the following: (a) age between 25 and 60years; (b) diagnosis of insulin resistance [homeostasis model assessment of insulin resistance (HOMA-IR) ≥2.6] (Garmendia et al., 2009); (c) to be physically inactive (i.e., a total of <150min/week of low-moderate physical activity or<75min/week of vigorous activity according to an International Physical Activity Questionnaire; O’Donovan et al., 2010); (d) to do not report exercise training participation the last 3months; (e) family history of T2DM; (f) to live in urban areas (related to modern life); (g) absence of musculoskeletal disorders; (h) absence of bone inflammatory, ischemic, and/or cardiac diseases; (i) absence of asthma and/or chronic obstructive pulmonary disease; (j) not to be under pharmacological treatment that modulates metabolic and/or respiratory control; and (k) free from hypertension and/or hypothyroidism diagnosis.

### Subjects

**Figure 1:**
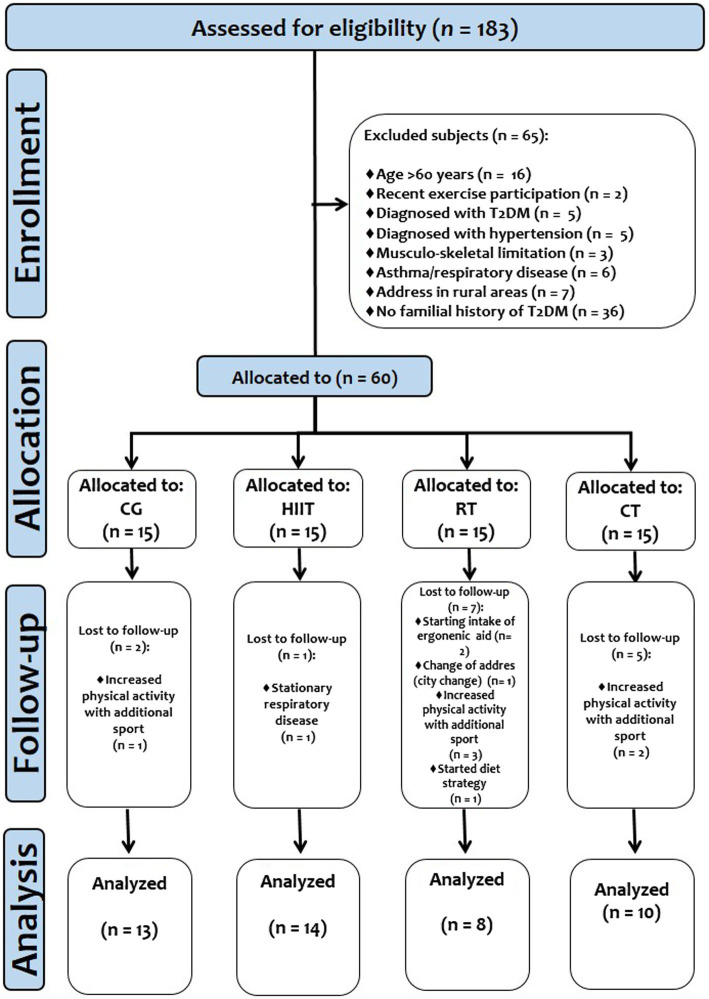
Study design.

After a public invitation from the Healthcare Center, 162 adult women were assessed for eligibility. A total of 82 individuals were excluded by: (a) age>60years (*n*=16), (b) reporting recent exercise participation (*n*=2), (c) hypertension diagnosis (*n*=5), (d) T2DM diagnosis (*n*=5), (e) hypothyroidism diagnosis (*n*=5), (f) musculoskeletal limitation (*n*=3), (g) no family history of T2DM (*n*=36), (h) stationary respiratory disease, such as asthma (*n*=6), and (i) inability to adhere to exercise sessions due to living in rural areas (*n*=4). Thus, from the 80 participants available, we extracted 5 that were randomly assigned 1:1 to control group (CG, *n*=20; HIIT, *n*=20; RT, *n*=20; and CT, *n*=20). Using a G*Power 3.1.9.7 statistical sample size calculator, with an *α* error probability 0.05, with 95% confidence interval (CI) for 4 groups, over three time points of measurements, and expecting medium-to-large effect sizes [(i.e., *η*^2^: from ≥0.06 to ≥0.14), a minimum of eight subjects per group would give a statistical power of ≥90%)]. A minimum of 70% compliance, and no intermittent adherence (do not adhere up to 4 or more continuous exercise sessions), was required to proceed with the final statistical analyses. Therefore, after follow-up, the final sample size analyzed was as follows: CG (*n*=13, age 40.0±11 y, BMI 28.3±3.6kg/m^2^), HIIT [*n*=14, age 37.0±9.0 y, BMI 28.6±3.6kg/m^2^, adherence mean 33 sessions (91%)], RT [*n*=8, age 34.0±9.0 y, BMI 29.4±5.5kg/m^2^, adherence mean 20 sessions (83%)], and CT [RT+HIIT; *n*=10, age 43.0±8.0 y, BMI 29.1±3.0kg/m^2^, adherence mean 30 sessions (83%)]. The study design is shown in Figure 1. The characteristics of the participants are shown in Table 1.

**Table 1:** Baseline characteristics of the study cohort assigned to the exercise or control groups.

Outcomes	Groups				Baseline *F*(), value of *p*, effect size (*η*^2^)
	CG^a^	HIIT^b^	RT^c^	CT^d^	
(*n*)	13	14	8	10	
Age (y)	40.0±11.0	37.0±9.0	34.0±9.0	43.0±8.0	*F*_(1.42)_, *p* =0.249, 0.09
Height (m)	1.57±0.05	1.55±0.04	1.58±0.05	1.55±0.04	*F*_(1.56)_, *p* =0.213, 0.10
**Body composition/Anthropometric**					
Body mass (kg)	69.8±10.5	68.4±7.9	74.6±17.9	69.8±7.5	*F*_(0.19)_, *p* =0.889, 0.02
Body mass index (kg/m^2^)	28.3±3.6	28.6±3.6	29.4±5.5	29.1±3.0	*F*_(0.19)_, *p* =0.913, 0.01
Skeletal muscle mass (%)	24.1±2.7	24.1±1.6	24.2±1.9	24.2±1.7	*F*_(0.02)_, *p* =0.994, 0.003
Humeral diameter (mm)	6.7±0.9	6.2±0.4	7.1±0.7	6.6±0.8	*F*_(2.41)_, *p* =0.083, 0.17
Femoral diameter (mm)	9.5±1.3	9.1±0.7	9.3±1.0	9.4±0.4	*F*_(0.57)_, *p* =0.639, 0.64
Arm perimeter (cm)	32.0±2.0	33.0±3.0	31.0±0.4	33.0±2.0	*F*_(0.83)_, *p* =0.489, 0.08
Calf perimeter (cm)	37.5±4.2	37.3±2.5	36.7±2.2	38.5±2.4	*F*_(0.27)_, *p* =0.845, 0.03
**Adiposity measures**					
Waist circumference (cm)	95.7±9.2	99.6±7.3	100.6±15.5	98.2±5.7	*F*_(0.31)_, *p* =0.811, 0.03
Tricipital skinfold (mm)	24.8±3.4	24.5±2.6	12.5±14.7	20.9±6.3	*F*_(1.16)_, *p* =0.341, 0.11
Subscapular skinfold (mm)	30.2±2.6	31.9±6.9	26.1±11.7	26.6±7.9	*F*_(1.17)_, *p* =0.336, 0.11
Ilio-cristale skinfold (mm)	31.0±3.0	32.4±4.3	31.0±10.9	24.7±5.7^b^	*F*_(0.45)_, ***p* =0.030**, 0.27
Calf skinfold (mm)	19.8±10.8	18.4±5.5	23.0±11.4	20.2±7.4	*F*_(0.34)_, *p* =0.791, 0.03

Data are shown as mean and±SD. Groups are described as: Control group (CG), High-intensity interval training (HIIT), Resistance training (RT), and Concurrent training (CT). Bold values denote significant pre-post changes at *p*<0.05 by repeated evaluations group × time. Univariant analysis was applied to test baseline differences among groups denoted by Sidak’s post-hoc (a, b, c, or d). values of *p* for significant differences are shown in bold. *η*^2^ denotes Lakens effect size.

### Testing Procedures

**Figure 2:**
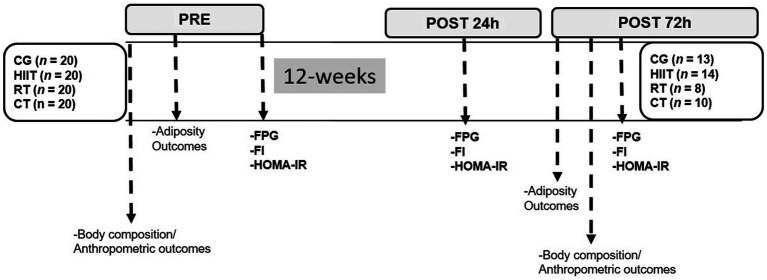
Study protocol. Groups are described as control group (CG), high-intensity interval (HIIT), resistance training (RT), and concurrent training (CT). Outcomes are described as fasting plasma glucose (FPG), fasting insulin (FI), and homeostasis model assessment on insulin resistance (HOMA-IR).

We measured FPG and fasting insulin (FI) to calculate HOMA-IR (Matthews et al., 1985). Blood samples (~3.5ml) were taken between 8 and 10h in the morning, after a 10-h overnight fast, in tubes with anticoagulant gels and placed on ice. Samples were then centrifuged at 4000rpm. (1700×*g*) for 5min at 4°C. Plasma samples were immediately transferred to pre-chilled microtubes and stored at −20°C for later analysis. FPG was analyzed enzymatically, as described (Alvarez et al., 2016), using standard kits (Wiener Lab Inc., Rosario, Argentina) on an automatic analyzer (Metrolab 2,300 Plus^™^, Metrolab Biomed Inc., Buenos Aires, Argentina). FI was measured by radio-immune assay (DPC, Los Angeles, CA, United States), as described (Álvarez et al., 2012). HOMA-IR was calculated using the equation: HOMA-IR=[FPG (mg/dl)×FI (μUI/dl)]/405; Matthews et al., 1985). FPG, FI, and HOMA-IR were assessed at three different points: at baseline (pre), at 24-h, and at 72-h after the last session of exercise or follow-up. We calculated the FPG, FI, and HOMA-IR delta changes from pre- to post-24h (∆FPG_24h_, ∆FI_24h_, and ∆HOMA-IR_24h_) and from post-24h to post-72h (∆FPG_72h_, ∆FI_72h_, and ∆HOMA-IR_72h_) of the last exercise session. All laboratory analyses were carried out at the Riñihue Private Clinic (Los Lagos, Chile). The study protocol is shown in (Figure 2).

### Anthropometric and Body Composition Measurements

Body mass was assessed by a digital weight scale with an accuracy of 0.1kg (Omron HBF-INT^™^, Omron Healthcare Inc., Lake Forest, IL, United States). Height was assessed with a stadiometer (Health o Meter^™^ Professional, Sunbeam Products Inc., Chicago, IL, United States) with an accuracy of 0.1cm. Body mass index (BMI) was calculated according to the formula: BMI=body mass (in kg)/(height)^2^ (in m). Waist circumference (WC) was measured using a flexible and inextensible measuring tape (Hoechstmass^™^, Sulzbach, Germany) with precision to the nearest 0.1cm. Skinfold was measured using a Langue^™^ skinfold caliper (Beta Technology Inc., Santa Cruz, California, United States). Using the pre- vs. post-24h, and post-24h vs. post-72h time comparisons, we calculated the delta changes (∆) as follows; delta waist circumference (∆WC in cm), with delta tricípital (∆T_SF_), delta subscapular (∆SE_SF_), delta ilio-cristale (∆IC_SF_), and delta calf skinfold (∆Calf_SF_) in mm. Skeletal muscle mass (SMM) was measured using a bioelectrical impedance analyzer (BIA) on a digital scale (Omron HBF-INT^™^, Omron Health Inc., Lake Forest, IL, United States), with the participants asked not to wear metal, watches, or jewelry (Álvarez et al., 2019). The BIA contains a foot, heel, and grip electrode and requires the user to introduce information for age (10–80y), sex (male/female), and height (100–199.5cm) of the participant. Thus, each participant in standing position holds the hand segment of the equipment for 30s approximately at an angle of 90° concerning the ground segment. Diameters of humeral (HumD), femoral (FemD), arm (ArmP), and calf (CalfP) perimeters were measured by using a small sliding bone caliper (Harpenden^™^, Canada) with 0.2mm graduation and 0–80mm range for measurement (Dykes et al., 1976). All outcomes were measured three times, with the average value registered. To the BIA analyses, all subjects were advised about to do not use metal jewelry, as well as to avoid abundant water 1h before the measurement. All the skinfold measurements were carried out by the same evaluator at pre- and post-test.

### High-Intensity Interval Training Program

HIIT was performed three times weekly (36 sessions) for 12-weeks, using a cycle ergometer (OXFORD^™^, model BE2601, Inc., Santiago, Chile). All participants received seven familiarization sessions before practicing HIIT. The participants performed a range of 8–12high-intensity cycling intervals of 60s, interspaced with 120s of passive recovery (seated on the bicycle and no moving). The rest period decreased progressively (120s in weeks 1–2, 105s in weeks 3–5, 90s in weeks 6–8, and 75s in weeks 9–10, and 60s in the final 12th week). Cycling revolutions at HIITs were maintained at 50 to 70rpm., representing 20 to 40km/h of speed. Subjects were required to cycle between 8 and 10 points on the modified Borg scale during the work interval (Gillen et al., 2013). This intensity corresponded to 80–100% of the maximum heart rate according to a described formula (Karvonen and Vuorimaa, 1988).

### Resistance Training Program

Using free weights with metal bars, participants developed four RT exercises, with a frequency of two times per week. However, they had a total of five exercise options and were required to swap between an upper body (first) and a lower body exercise, as follows: 1. biceps curl, 2. half squat, 3. shoulder press, 4. calf raises, and 5. tricípital flexion/extension, plus two optional preventive exercises developed only by using their body weight (abdominal crunches and lower back concentric exercise). All participants received seven familiarization sessions before the RT intervention. Progressive RT programs were performed three times per week for 12-weeks. The RT program consisted of an interval of work (performing voluntary extension/contraction) during 60s in which a range of 8–10 points of the modified Borg scale was reached in the final 55–60s. The intensity in terms of 1RM corresponded to 20–40% at baseline and between 25 and 50% of the 1RM at the post-test. The recovery period was 120s between sets. Each interval of work was repeated three times in the four exercises. The total intervals of work were 12.

### Concurrent Training Program

This program consisted of 3 weekly sessions and included RT first followed by HIIT exercises secondly, such as cycling sprints [for 40 (RT) and 20 (HIIT) minutes]. This choice was based on previous research experience for to be more efficient the exercise on health markers (Delgado-Floody et al., 2021). The number and types of RT exercises applied in the CT protocol included the same exercises as the RT program alone, which was performed with dumbbells, free weights, and metal bars, plus the two optional preventive exercises previously mentioned. Each RT exercise consisted of 60s of concentric/eccentric voluntary movements, interspersed by 60s of recovery. In the HIIT program, the participants performed a range of 8–12high-intensity cycling intervals of 60s, interspaced with 120s of passive recovery (seated on the bicycle and no moving). The rest period decreased progressively (120s in weeks 1–2, 105s in weeks 3–5, 90s in weeks 6–8, 75s in weeks 9–10, and 60s in the final 12th week), in a similar protocol (i.e., volume and intensity) as was described in the HIIT program section. As additional information, and after intervention, the delta in percentage of 1RM changes (∆_%1RM_) was very similar between CT vs. RT by exercise; biceps curl ∆_%1RM_+50.0% vs. +25.0%, half squat ∆_%1RM_+18.7% vs. +18.7%, shoulder press ∆_%1RM_+70.0% vs. +70.0%, gastrocnemius ∆_%1RM_+210.0% vs. +210.0%, and triceps flexion/extension ∆_%1RM_+28.0% vs. +30.0% (data not shown).

For the HIIT and RT exercise sessions, subjects adhered to spend 45kcalkg^−1^·min^−1^ energy expenditure per session (HIIT; 12min, and RT; 12min, and cool-down/session ~24min), which was equivalent to ~540kcal of expended energy per week; the CT sessions were based on 90kcal·kg^−1^·min^−1^ energy expenditure per session (24min, and cool down/session ~48min), which was equivalent to ~1,080kcal of expended energy per week at the end of the training.

Each HIIT, RT, and CT program were performed in the morning from 9 to 12h.

### Residual Effects of HIIT, RT, and CT Post-72-H of Training Cessation

We applied the following two analyses: (a) pre- vs. post-24h (i.e., for training-induced changes) and (b) post-24h vs. post-72h for FPG and FI, and including the calculated HOMA-IR. We calculated the delta changes values (∆) from the absolute values of FPG and FI. This resulted in (a) pre- vs. post-24h measurement for the outcomes ∆FPG_24h_, and ∆FI_24h_, and (b) post-24h vs. post-72h analyses, measuring the ∆FPG_72h_, and ∆FI_72h_ outcomes. The residual effect of the CT was extracted from the delta changes of the analyses of ∆FPG_72h_ and ∆FI_72h_ outcomes. All subjects were advised not to consume caffeine meals before and after the metabolic measurements.

After this procedure, we correlated the outcomes ∆FPG_72h_, ∆FI_72h_ with anthropometric and body composition outcomes generated from the 12-week interventions in each HIIT, RT, and CT regimen including delta change of skeletal muscle mass (∆SMM), humeral diameter (∆HumD), femoral diameter (∆FemD), arm perimeter (∆ArmP), and calf perimeter (∆CalfP).

Before starting the measurements of metabolic outcomes FPG, FI, and HOMA-IR at baseline, the basal metabolic rate information was delivered to each subject participant of the four groups (mean of basal metabolic rate baseline was of HIIT: 1307kcal/day, RT: 1403kcal/day, CT: 1291kcal/day, and CG: 1318kcal/day, according to with baseline age, height, weight, and sex). Thus, we recorded this information a post-test 24 and 72h of the exercise cessation, in order to maintain these baseline patterns.

### Statistical Analysis

Data are presented as the mean±standard deviation (in tables and figures). For the main outcomes, delta changes from pre- to post-24h were calculated for FPG, FI, and HOMA-IR (∆FPG_24h_, ∆FI_24h_, and HOMA-IR_24h_), and from post-24h to post-72h (∆FPG_72h_, ∆FI_72h_, and HOMA-IR_72h_). For secondary outcomes, delta changes were calculated only from pre- to post-24h. The normality and homoscedasticity assumptions were tested by the Shapiro-Wilk test and Levene’s test (*F*), respectively. The Wilcoxon test was used for non-parametric data per group (Main outcomes; FI in HIIT, RT, and CT; HOMA-IR in HIIT and RT group, secondary outcomes; and Age in RT, SMM in CG, calf perimeter in CG, and humeral diameter in CT group). A repeated-measures ANOVA [groups×time (Pre–Post_24h_–Post_72h_)] was used to assess training-induced effects for the main metabolic outcomes (i.e., FPG, FI, and HOMA-IR) after 12-weeks of intervention, and all those outcomes measured at Pre-Post_24h_. Subsequently, delta change values were calculated for the main and secondary outcomes, and Sidack’s *post-hoc* test was applied when the *F* value was significant. The Eta partial squared statistic for interaction (Time×Group) was assessed by *η*^2^ obtained from the Univariant test with small (*η*^2^=0.01), medium (*η*^2^=0.06), and large (*η*^2^=0.14) effects defined according to Lakens (2013). An ANCOVA was conducted in adjustments for the baseline FPG values with age and BMI as covariates. After this, we applied a linear regression model to predict the ∆FPG_72h_ and ∆FI_72h_ changes using body composition, anthropometric, and adiposity outcomes, after using a multicollinearity test of tolerance and variance inflation. Statistical analyses were performed with SPSS statistical software version 18 (SPSS^™^ Inc., Chicago, Illinois, United States), and the graphs/figures were designed in GraphPad Prism version 8.0 version (USA) software. The alpha level was fixed at *p*≤0.05 for all statistical significance.

## Results

### Main Outcomes: Training-Induced Effects on FPG, Insulin, and HOMA-IR

The overall baseline data and training-induced effects for the metabolic outcomes FPG, FI, and HOMA-IR are shown in Table 2.

**Table 2:** Changes in plasma markers of insulin-resistant adult women, participants of 12-weeks of high-intensity interval, resistance, concurrent training, or a control group.

Outcomes	Time	CG^a^	HIIT^b^	RT^c^	CT^d^
(*n*=)		13	14	8	10
Fasting plasma glucose (mg/dl)	Pre	102.0±9.0	101.0±0.9	98.0±13.0	99.0±5.0
	Post_24h_	103.1±9.4	95.3±12.1^**^	92.9±9.6	96.7±4.4
	Post_72h_	103.4±8.8	96.5±12.8	93.9±5.6	103.3±6.1^***^
Fasting insulin (μUI/dl)	Pre	3.6±1.7	4.6±2.9	4.9±4.6	4.5±4.8
	Post_24h_	3.7±1.7	4.9±5.0	4.3±4.1	4.7±4.3
	Post_72h_	3.8±1.7	4.4±2.8	2.8±2.0	4.5±4.2
HOMA-IR	Pre	0.9±0.5	1.2±0.8	1.3±1.5	1.1±1.1
	Post_24h_	1.0±0.5	1.2±1.2	1.1±1.2	1.1±1.1
	Post_72h_	1.0±0.5	1.1±0.7	0.6±0.2	1.2±1.2

** Significant differences from pre- to post-24h by two-way ANOVA (group×times) at *p*<0.001.

*** Significant differences from post-24h to post-72h by two-way ANOVA (Group×Time) at *p*<0.001.

Data are shown as mean and±SD. Groups are described as: Control group (CG), High-intensity interval (HIIT), Resistance training (RT), and Concurrent training (CT). Bold values denote significant pre-post changes at *p*<0.05 by repeated evaluations×group. Univariant analysis was applied to test baseline differences among groups denoted (a, b, c, or d). value of *p* significant differences was remarked in bold.

### Main Outcomes: Residual Effects on Metabolic Outcomes Post-24H

**Figure 3:**
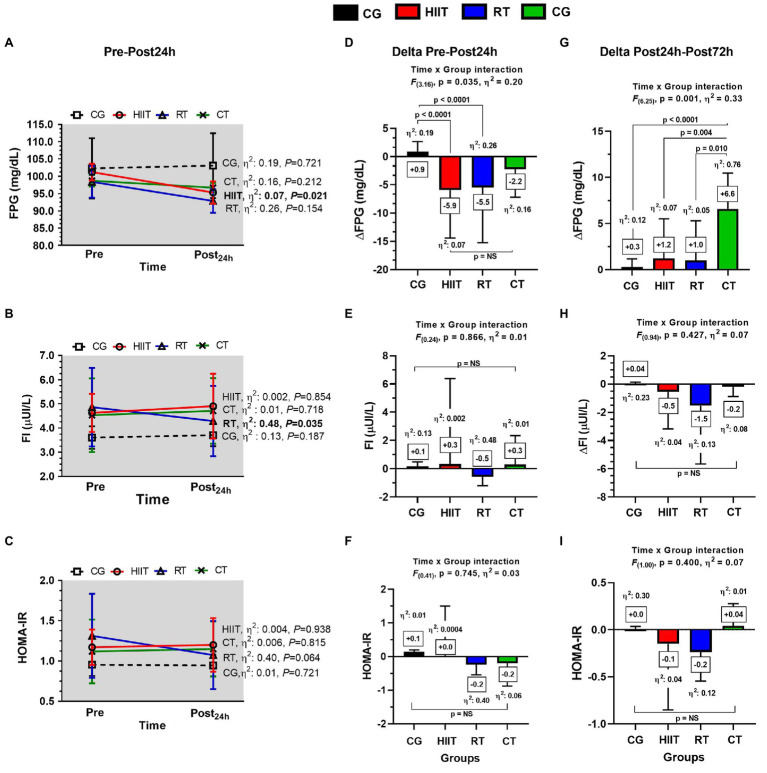
Long-term **(A-C)** and acute post-24h **(D-F)** and post-72h changes **(G-I)** in metabolic outcomes in adult women with insulin resistance after 12-weeks of the three different exercise trainings or a control group. Panels **(A-C)** show values in mean±SEM during the exercise period. Panels **(D–F)** show values in “delta changes” (from pre- to post-24h). Panels **(G–I)** show values in “delta (Δ) changes” (from post-24h to post-72h). Groups are described as: control group (CG), high-intensity interval training (HIIT), resistance training (RT), and concurrent training (CT). Outcomes are described as: fasting plasma glucose (FPG), fasting insulin (FI), and homeostasis model assessment of insulin resistance (HOMA-IR). ∆FPG_24h_, ∆FI_24h_, and ∆HOMA-IR_24h_ in panels **(D–F)**, respectively, denote deltas from pre- to post-test 24h after the last exercise session. ∆FPG_72h_, ∆FI_72h_, and ∆HOMA-IR_72h_ in panels **(G–I)**, respectively, denote deltas from post-24h to post-72h after the last exercise session. Within-group changes are described with specific at values of *p*≤0.05 in bold in **(A,D,G)**. Between-group changes are described in bold values in panels **(D–I)**.

At training-induced changes, the HIIT intervention resulted in a marked decrease in FPG when comparing pre- vs. post-condition [mean±SD, effect size (*η*^2^), value of *p*, in forward], FPG 101.0±2 vs. 95.3±3.2mg/dl, *η*^2^: 0.07, *p*=0.021; Figure 3A). FI significantly decreased after the RT intervention from pre- to post-test (4.9±1.6 vs. 4.3±1.5 μUI/L, *η*^2^: 0.48, *p*=0.035; Figure 3B), and no significant changes were observed for HOMA-IR (Figure 3C).

At residual effects, in FPG, at the 24h, they were significant time×group interaction among groups [*F*_(3.16)_, *η*^2^: 0.20, *p*=0.035], with significant differences in the delta (∆) effects between HIIT ∆FPG_24h_−5.9 vs. CG+0.9mg/dl, *η*^2^: 0.19, *p*<0.0001, and between RT ∆FPG_24h_−5.5 vs. CG+0.9mg/dl, *η*^2^: 0.26, *p*<0.0001, but no differences were found between HIIT, RT, and CT (Figure 3D). At 24h, no time×group interactions were found for FI (Figure 3E) and HOMA-IR (Figure 3F).

### Main Outcomes: Residual Effects on Metabolic Outcomes Post-72H

In FPG, at the 72h, they were significant time×group interaction among groups [*F*_(6.25)_, *η*^2^: 0.33, *p*=0.001], with significant differences in the delta (∆) effects between CT ∆FPG_72h_+6.6 vs. CG+0.3mg/dl, *η*^2^: 0.76, *p*<0.0001, between CT ∆FPG_72h_+6.6 vs. HIIT+1.2mg/dl, *η*^2^: 0.07, *p*=0.004, and between CT ∆FPG_72h_+6.6 vs. RT 1.0mg/dl, *η*^2^: 0.05, *p*=0.010 (Figure 3G), but no interactions were found for FI (Figure 3H) and HOMA-IR (Figure 3I).

### Secondary Outcomes: Body Composition and Anthropometric Markers Post-12-Weeks

**Figure 4:**
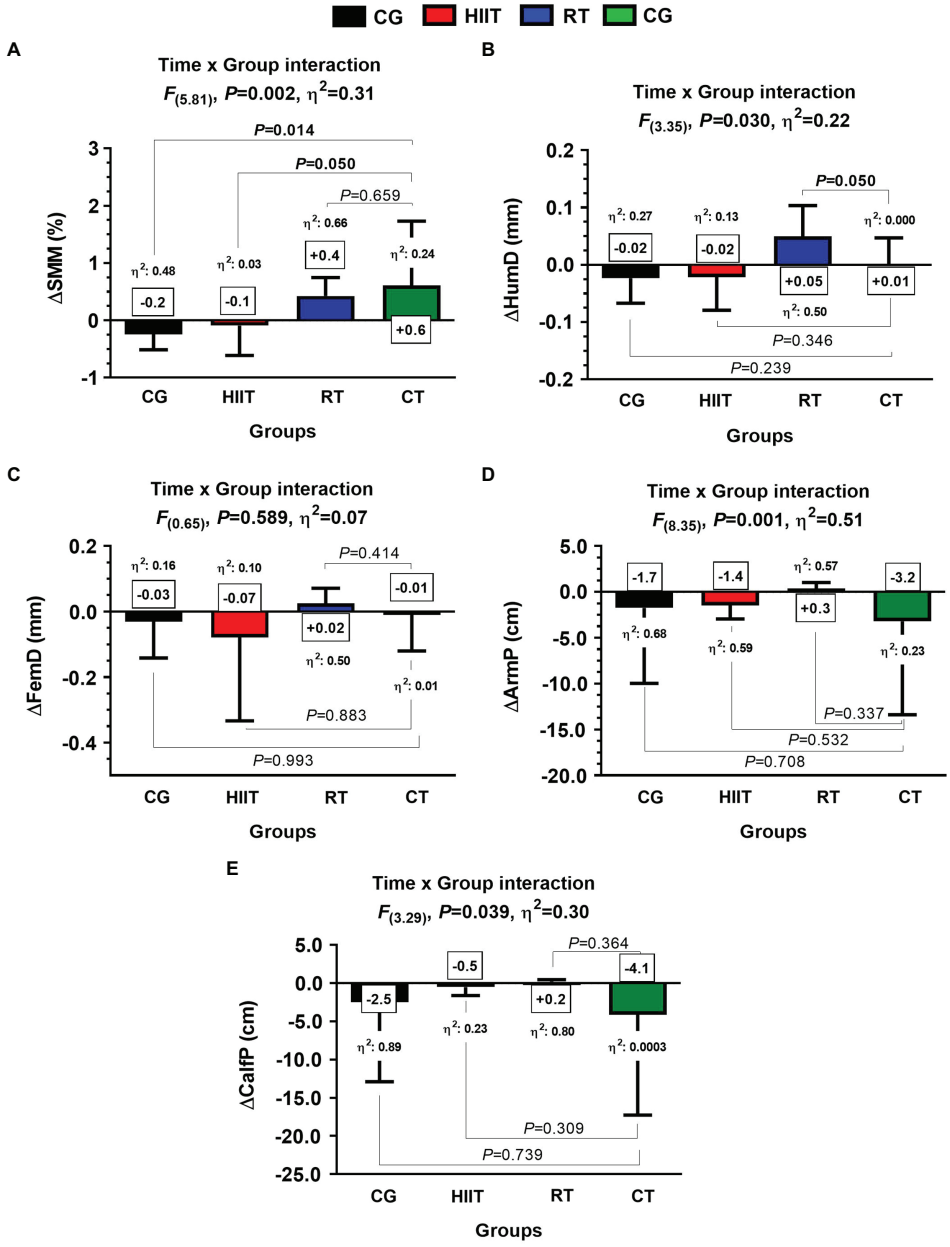
Body composition and anthropometric adaptations in skeletal muscle markers after 12-weeks of three different exercise training programs, or in a control group, in adult women with insulin resistance. Groups are described as: control group (CG), high-intensity interval training (HIIT), resistance training (RT), and concurrent training (CT) group. Outcomes are described as delta skeletal muscle mass (∆SMM) expressed in kg, delta humeral diameter (∆HumD), delta femoral diameter (∆FemD), delta arm perimeter (∆ArmP), and delta calf perimeter(∆CalfP). Between-group changes are described in bold values in panels **(A,B)**. *η*^2^ denotes effect size.

In SMM, they were significant time×group interaction among groups [*F*_(5.81)_, *η*^2^: 0.31, *p*=0.002], with significant differences in the delta (∆) effects between CT ∆SMM+0.6 vs. CG+0.2kg, *η*^2^: 0.48, *p*=0.014 and between CT ∆SMM+0.6 vs. HIIT −0.1kg, *η*^2^: 0.03, *p*=0.050 (Figure 4A). In the ∆HumD, there were significant time×group interaction among groups [*F*_(3.35)_, *η*^2^: 0.22, *p*=0.030], with significant differences in the delta (∆) effects between RT ∆HumD+0.05 vs. CT+0.01mm, *η*^2^: 0.000, *p*=0.050 (Figure 4B). No time×group interaction was observed in FemD (Figure 4C). In ∆ArmP, and despite that they were observed significant time×group interaction among groups [*F*_(8.35)_, *η*^2^: 0.51, *p*=0.001], no significant differences among the delta (∆) effects were found between CT vs. HIIT, RT, or CG, (Figure 4D). No time×group interaction was observed in CalfP (Figure 4E).

### Secondary Outcomes: Adiposity Markers Post-12-Weeks

**Figure 5:**
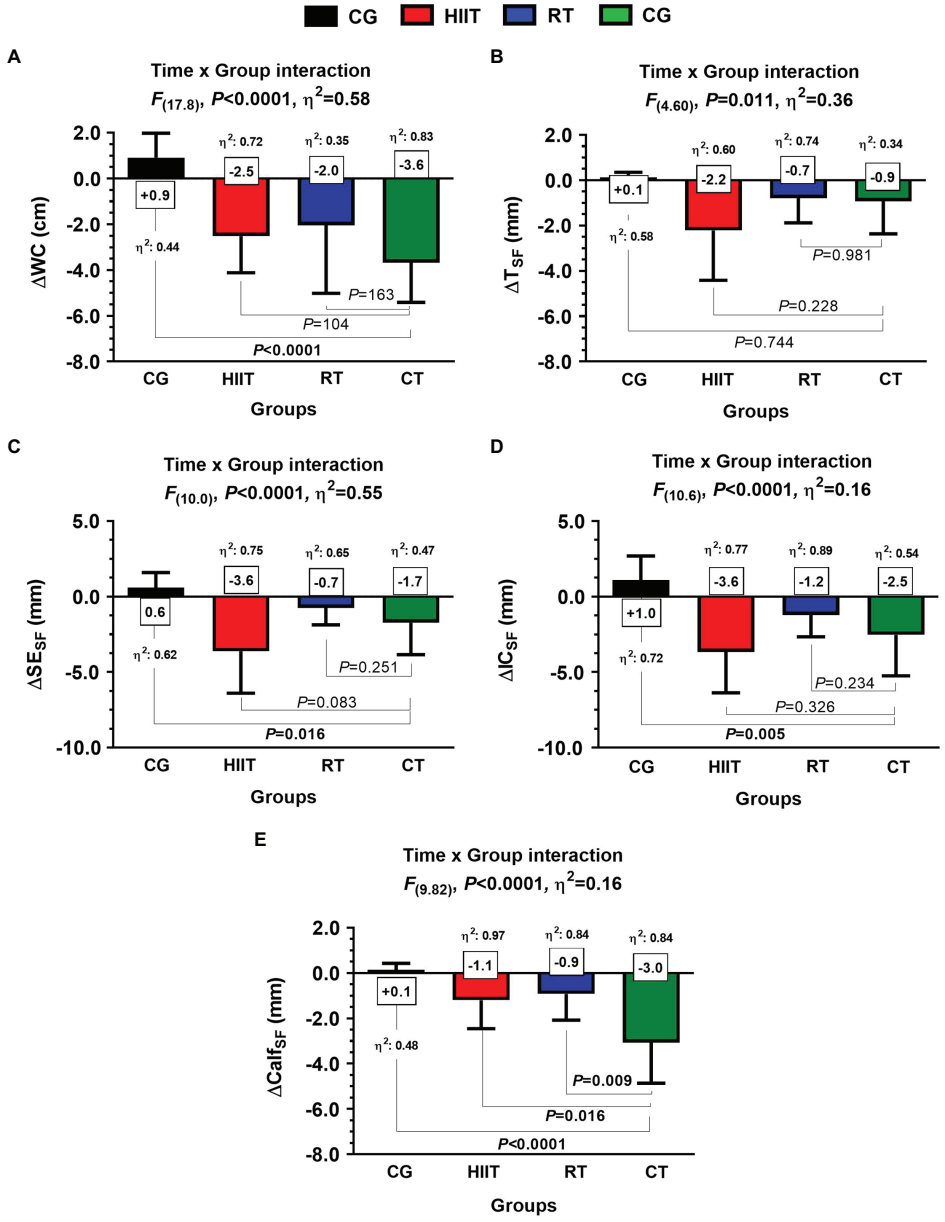
Adaptations of adiposity markers after 12-weeks of three different exercise training programs, or in a control group, in adult women with insulin resistance. Groups are described as: control group (CG), high-intensity interval training (HIIT), resistance training (RT), and concurrent training (CT). Outcomes are described as delta waist circumference (∆WC), delta tricípital skinfold (∆T_SF_), delta subscapular skinfold (∆SE_SF_), delta ilio-cristale skinfold (∆IC_SF_), and delta calf skinfold (∆Calf_SF_). Panel A show waist circumference, panel B tricícital skinfold, panel C subescapular skinfold, panel D ilio-crestale skinfold, and panel E calf skinfold. Between-group differences are described in bold values from panels **(A–E)**. *η*^2^ denotes effect size.

In WC, they were significant time×group interaction among groups [*F*_(17.8)_, *η*^2^: 0.58, *p*<0.0001], with significant differences in the delta (∆) effects between CT ∆WC –3.6 vs. CG+0.9cm, *η*^2^: 0.44, *p*<0.0001, (Figure 5A). In T_SF_, despite they were significant time×group interaction among groups [*F*_(4.60)_, *η*^2^: 0.36, *p*=0.011], no differences were found among the delta (∆) effects between CT vs. HIIT, RT, or CG (Figure 5B). In SE_SF_, there were significant time×group interaction among groups [*F*_(10.0)_, *η*^2^: 0.55, *p*<0.0001], with significant differences in (∆) effects between CT ∆SE_SF_ –1.7 vs. CG+0.6mm, *η*^2^: 0.62, *p*=0.016 (Figure 5C). In IC_SF_, there were significant time×group interaction among groups [*F*_(10.6)_, *η*^2^: 0.16, *p*<0.0001], with significant differences in (∆) effects between CT ∆IC_SF_ –2.5 vs. CG+1.0mm, *η*^2^: 0.72, *p*=0.005 (Figure 5D). In Calf_SF_, there were significant time×group interaction among groups [*F*_(9.82)_, *η*^2^: 0.16, *p*<0.0001], with significant differences in (∆) effects between CT ∆Calf_SF_ –3.0 vs. CG+0.1mm, *η*^2^: 0.48, *p*<0.0001, between CT ∆Calf_SF_ –3.0 vs. HIIT −1.1mm, *η*^2^: 0.97, *p*=0.016, and between CT ∆Calf_SF_ –3.0 vs. RT −0.9mm, *η*^2^: 0.84, *p*=0.009 (Figure 5E).

### Training-Induced Changes From CT for Predicting Residual Effects on FPG and Insulin Changes Post-72H

Using a linear regression model and considering the delta changes in ∆FPG_72h_, 4 out of 5 adaptations, such as body composition/anthropometry [model 1 (30.8%) ∆SMM, *r*=−0.55, *p*<0.001]; model 2 (51.0%) ∆SMM+∆HumD, *r*=−0.50, *p*=0.002; model 3 (57.6%) ∆SMM+∆HumD+∆FemD, *r*=0.21, *p*=0.004; and model 4 (62%) ∆SMM+∆HumD+∆FemD+∆ArmP, *r*=0.62, *p*=0.015; Table 3), as well as adiposity adaptations [model 1 (17.5%) ∆WC, *r*=0.41, *p*=0.009]; model 2 (28.3%) ∆WC+∆T_SF_, *r*=−0.22, *p*=0.014; and [model 3 (28.7%) ∆WC+∆T_SF_+∆SE_SF_, *r*=0.05, *p*=0.037], predicted in different percentages (i.e., from 17.5 to 62.6%) the FPG_72h_ in the CT group (Table 3).

**Table 3:** Body composition/anthropometric and adiposity outcomes (delta changes) predicting the changes in fasting plasma glucose from post-24h to post-72h in participants after 12-weeks of concurrent training.

Models for predicting ∆FPG_72h_	*R*^2^ (% of Prediction)	β (95%CI)	*r*=	Model value of *p*
**Body composition/anthropometry outcomes**				
Model 1	0.308 (**30.8%**)^†^	−0.55 (−4.4, 0.8)	−0.55	***p* <0.001**
Model 2	0.510 (**51.0%**)^†^	−0.46 (−4.1, 1.1)	−0.50	***p* =0.002**
Model 3	0.576 (**57.6%**)^†^	−0.55 (−4.9, 1.3)	0.21	***p* =0.004**
Model 4	0.626 (**62.6%**)^†^	−0.38 (−6.0, 3.5)	0.62	***p* =0.015**
Model 5	0.640 (64.0%)	−0.53 (−12.5, 9.1)	0.24	*p* =0.069
**Adiposity outcomes**				
Model 1	0.175 (**17.5%**)^†^	0.41 (−0.80, 2.51)	0.41	***p* =0.009**
Model 2	0.283 (**28.3%**)^†^	0.49 (−0.76, 2.79)	−0.22	***p* =0.014**
Model 3	0.287 (**28.7%**)^†^	0.50 (−1.02, 3.08)	0.05	***p* =0.037**
Model 4	0.312 (31.2%)	0.46 (−1.55, 3.45)	0.23	*p* =0.061
Model 5	0.468 (46.8%)	0.32 (−2.39, 3.73)	0.23	*p* =0.089

† denotes models that predict significantly the changes in FPG post-72h of training cessation.

Delta fasting plasma glucose (∆FPG_72h_) from post-24h to post-72h. Outcomes are described as: delta skeletal muscle mass (∆SMM) expressed in percentage, delta humeral diameter (∆HumD), delta femoral diameter (∆FemD), delta arm perimeter (∆ArmP), and delta calf perimeter (∆CalfP). delta waist circumference (∆WC), delta tricípital skinfold (∆T_SF_), delta subscapular skinfold (∆SE_SF_), supra-iliac skinfold (∆SI_SF_), and delta calf skinfold (∆Calf_SF_). Body composition/anthropometric markers were adjusted by (Model 1) ∆SMM non-adjusted model, (Model 2) ∆SMM adjusted by ∆HumD, (Model 3) ∆SMM adjusted by ∆HumD and ∆FemD, (Model 4) ∆SMM adjusted by ∆HumD, ∆FemD, and ∆ArmP, and (Model 5) ∆SMM adjusted by ∆HumD, ∆FemD, ∆ArmP, and ∆CalfP. Adiposity markers were adjusted by (Model 1) ∆WC non-adjusted model, (Model 2) ∆WC adjusted by ∆T_SF_, (Model 3) ∆WC adjusted by ∆T_SF_ and ∆SE_SF_, (Model 4) ∆WC adjusted by ∆T_SF_, ∆SE_SF_, and ∆IC_SF_ and (Model 5) ∆WC adjusted by ∆T_SF_, ∆SE_SF_, ∆IC_SF_, and ∆Calf_SF_. Bold values denote significant association at *p*<0.05 by different linear regression models; Pearson *r*-test (r).

On the other hand, the body composition/anthropometry was the unique model for predicting changes in ∆FI_72h_ after CT exercise intervention, by model 5 [(99.7%) ∆SMM+∆HumD+∆FemD+∆ArmP+∆CalfP, *r*=0.01, *p*=0.036] (Table 4).

**Table 4:** Body composition/anthropometric and adiposity outcomes (delta changes) predicting the changes in FI from post-24h to post-72h in participants after 12-weeks of concurrent training.

Models for predicting ∆FI_72h_	*R*^2^ (% of Prediction)	β (95%CI)	*r*=	Model value of *p*
**Body composition/anthropometry outcomes**				
Model 1	0.018 (1.8%)	−0.13 (−0.64, 0.51)	−0.13	*p* =0.799
Model 2	0.776 (77.6%)	0.04 (−0.30, 0.35)	0.88	*p* =0.379
Model 3	0.815 (81.5%)	0.11 (−0.32, 0.45)	0.03	*p* =0.287
Model 4	0.988 (98.8%)	0.21 (−0.29, 0.03)	0.54	*p* =0.071
Model 5	0.997 (**99.7%**)^†^	−0.08 (−0.23, 0.13)	0.01	***p* =0.036**
**Adiposity outcomes**				
Model 1	0.079 (7.9%)	−0.28 (−0.44, 0.22)	−0.28	*p* =0.316
Model 2	0.137 (13.7%)	−0.33 (−0.50, 0.24)	0.17	*p* =0.369
Model 3	0.196 (19.6%)	−0.36 (−0.56, 0.27)	0.14	*p* =0.573
Model 4	0.266 (26.6%)	−0.29 (−0.61, 0.37)	−0.19	*p* =0.583
Model 5	0.286 (28.6%)	−0.34 (−0.81, 0.54)	0.19	*p* =0.870

† denotes models that predict significantly the changes in FI post-72h of training cessation.

Delta fasting insulin (∆FI_72h_) from post-24h to post-72h. Outcomes are described as: delta skeletal muscle mass (∆SMM) expressed in percentage, delta humeral diameter (∆HumD), delta femoral diameter (∆FemD), delta arm perimeter (∆ArmP), and delta calf perimeter (∆CalfP). delta waist circumference (∆WC), delta tricipital skinfold (∆T_SF_), delta subscapular skinfold (∆SE_SF_), supra-iliac skinfold (∆SI_SF_), and delta calf skinfold (∆Calf_SF_). Body composition/anthropometric markers were adjusted by (Model 1) ∆SMM non-adjusted model, (Model 2) ∆SMM adjusted by ∆HumD, (Model 3) ∆SMM adjusted by ∆HumD and ∆FemD, (Model 4) ∆SMM adjusted by ∆HumD, ∆FemD, and ∆ArmP, and (Model 5) ∆SMM adjusted by ∆HumD, ∆FemD, ∆ArmP, and ∆CalfP. Adiposity markers were adjusted by (Model 1) ∆WC non-adjusted model, (Model 2) ∆WC adjusted by ∆T_SF_, (Model 3) ∆WC adjusted by ∆T_SF_ and ∆SE_SF_, (Model 4) ∆WC adjusted by ∆T_SF_, ∆SE_SF_, and ∆IC_SF_ and (Model 5) ∆WC adjusted by ∆T_SF_, ∆SE_SF_, ∆IC_SF_, and ∆Calf_SF_. Bold values denote significant associations at *p*<0.05 by different linear regression models; Pearson *r*-test (*r*).

## Discussion

The present study aimed to assess the residual effects (post-72-h training cessation) on FPG and FI after 12-weeks of HIIT, RT, or CT in women with IR. We also aimed to determine the training-induced, post-training residual impact of CT. The main findings were as follows: (i) HIIT decreases FPG and RT decreases FI 24-h post-training, (ii) both HIIT and RT exercises alone show improved residual effects on FPG and FI (post-72h) compared with the CT intervention where at this time their effects are worsened, and (iii) adiposity-related markers [model 2: ∆WC+∆T_SF_, *R*^2^ 0.62 (62%)] together with body composition/anthropometric adaptations [model 4: ∆SMM+∆HumD+∆FemD+∆ArmP, *R*^2^ 0.28 (28%)] models mostly explain the poorer residual effects promoted by the CT intervention. These results highlight that both adiposity reduction particularly, together with SMM, and bone increases play a particular role in the residual effects after CT.

While there is a large body of evidence on the training-induced changes in metabolic markers after HIIT (Little et al., 2011; Jelleyman et al., 2015; Alvarez et al., 2016; Matos et al., 2018), RT (Dunstan et al., 2002; Wood and O’Neill, 2012), and CT (Timmons et al., 2018; Álvarez et al., 2019), evidence is sparse concerning their residual effects post-exercise, particularly for CT. For instance, Marcus et al. (2013) showed that despite 16-weeks of RT in patients with T2DM, which promoted an improvement in SMM (∆ 8%) and regional insulin sensitivity based on a euglycemic-hyperinsulinemic clamp (∆ 33.9%), there was a loss in whole-body insulin sensitivity (∆ –12.4%) 7days after exercise csessation even though SMM was maintained. Also, when highly trained athletes and young people were participating under MICT, these showed showed an increase in FI and a decrease in insulin sensitivity after 5 to 7days post-training (Vukovich et al., 1996; Goulet et al., 2005). Contrastingly, the present study shows that after 12-weeks of HIIT, RT, or CT, only HIIT and RT groups showed improve ∆FPG_24 h_, and the RT group showed improved ∆FI_24h_. Nevertheless, our data reveal that improvements from HIIT in ∆FPG_24h_ were then worsened after 72h of exercise cessation (∆FPG_72h_+1.2mg/dl), from RT these worsened was from −5.5 at 24h to +1.0 at 72h, but more worryingly in CT from −2.0 at 24h to +6.6mg/dl at post-72h, which is similar to previous reports (Vukovich et al., 1996; Goulet et al., 2005). Accordingly, our data along with the aforementioned evidence suggest that HIIT, and RT alone, but also CT can decrease (i.e., significant or not) FPG particularly 24-h post-exercise cessation, but however is the CT regime that shows a minor efficiency for maintaining these beneficial residual effect post-72h of exercise cessation. These results open the door for speculating that the positive metabolic adaptations promoted during exercise training participation could be critically dependent on maintaining this regular exercise routine in this precise metabolic topic of IR patients, but it is a matter of future and more complex studies to corroborate these findings.

Regarding the physiological mechanisms related to anthropometric/body composition on metabolic control, it is well known that SMM plays an important role in glucose control through peripheral glucose uptake (Richter et al., 1988). In the present study, we observed minimal increases in SMM in the RT (∆SMM; +0.4%) and CT (∆SMM; +0.6%) groups (Table 1 and Figure 4A). Additionally, other outcomes could be involved in the beneficial residual effects on glucose control post-exercise, such as WC or subcutaneous fat decreases, as previously reported in IR cohorts. Other anthropometric effects on metabolic health are shown for example after 14-weeks of a) exercise plus weight loss or b) exercise without weight loss that led to a reduction in both ∆FPG −5.8 and−1.5mg/dl, and ∆FI –44.4 and −13.7%, respectively, in each strategy (Ross et al., 2004). However, considering the increases in SMM in RT and CT, but also the higher decreases in adiposity (i.e., WC and subcutaneous fat) markers after HIIT, it is possible to presume that the residual effect and thus their prolonged beneficial effects of exercise on glucose control in FPG can be highly dependent more on fat reduction than SMM increases post-exercise cessation. In brief, part of these presumptions has been previously described in the physiopathology of the IR (Abdul-Ghani and DeFronzo, 2010), and the athlete paradox, which is relevant to this exercise intervention in our IR cohort (Goodpaster et al., 2001). Following this, exercise training could decrease both adiposity markers as can be seen in Table 1 and Figure 5, and potentially also at intramyocellular content as previously (Dube et al., 2008), and thus indirectly prolong insulin sensitivity from the last exercise cession in both HIIT and RT.

The possibility of “interference effects” from CT adaptations has been proposed from the combination of RT (i.e., increased SMM and bone mass) or moderate-intensity CT (i.e., decrease in body fat markers) regimens, in contrast to the individual application of RT and moderate-intensity CT (Wilson et al., 2012; Lundberg et al., 2013; Fyfe et al., 2014). However, our data reveal that CT promoted a remarkable metabolic beneficial effect. Indeed, 12-weeks of CT modified FPG; however, this improvement was rapidly lost at 72-h post-training, increasing ∆FPG_72h_ (Δ +6.6mg/dl). Based on these results, CT exercise appears to be the intervention that most rapidly loses its acquired benefits post-training compared with the HIIT and RT regimens, showing the poorest residual capacity to maintain FPG from 24h to post-72h of exercise cessation. There is evidence that CT could compromise the adaptations induced by each HIIT and RT alone (Fyfe et al., 2014; Coffey and Hawley, 2017); it consists in that signaling responses mitochondrial biogenesis adaptations [i.e., adenosine monophosphate (AMP)-activated protein kinase (AMPK), Ca2+/calmodulin-dependent kinase II, and peroxisome proliferator-activated receptor-c coactivator-1] seem to diminish the muscle anabolic pathways activated by RT [i.e., mechanistic target of rapamycin complex 1 (mTORC1)] and downstream effectors the 70kDa ribosomal S6 protein kinase (S6K) and eukaryotic initiation factor 4E binding protein (4E-BP1; Ronnestad et al., 2012; Jones et al., 2016; Coffey and Hawley, 2017), and although we do not measure these molecular proteins, but apparently, it seems not at this moment to affect the metabolic and cardiovascular adaptations from CT (Álvarez et al., 2019; Delgado-Floody et al., 2020, 2021).

Training volume (quantity) has been proposed as one of the principles of training along with quality (intensity; Da Silva et al., 2020) and offers a possible explanation for our results. Our study used low frequency and volume training for RT and HIIT modalities, in contrast to the higher frequency and volume training for the CT modality (RT group: two sessions/week, 80min/week; HIIT group: three sessions/week, 60min/week; and CT group: three sessions/week, 180min/week). It is therefore difficult to speculate on the possible mechanisms for the low maintenance (i.e., low residual capacity) of FPG_72h_ in the CT intervention. The physiological effects of CT (combining RT and HIIT in this specific order) were then presumably neither synergic nor additive, as was shown by worsened in FPG from 24h to 72h (+6.6mg/dl). Moreover, we observed relative maintenance of both FPG and FI at 72h after the RT cessation but not after the combination with HIIT in CT. For example, Tokmakidis et al. (2014) reported that 9months of CT resulted in an ∆FPG −12% in patients with T2DM, whereas this outcome was increased slightly (+0.7%) 3months after exercise cessation; however, improvements in glycosylated hemoglobin were promoted by 9months of CT were completely reversed after 3months of physical inactivity. This information can also suggest that metabolic adaptations following a period of physical training are critically dependent on being regular and consistent.

Considering that CT is a combination of RT and HIIT, our data revealed that exercise adaptation for secondary endpoints of adiposity markers, the results for WC, T_SF_, SE_SF_, IC_SF_, and Calf_SF_ revealed that HIIT alone showed greater effect sizes for improving (i.e., decreasing) these markers, followed by RT, whereas CT showed the lowest effect size for these outcomes (Figure 5). The role of HIIT in stimulating molecular mechanisms that improve fat oxidation is well established (Astorino and Schubert, 2018), and catecholamines are highly activated after intermittent exercise, such as HIIT (Boutcher, 2011). Thus, one may speculate that additionally to those adiposity markers that were decreased post-exercise, other potential mechanisms underlying the residual effects post-HIIT and RT at least can be related with those acute hormonal roles from post-exercise cessation (Boutcher, 2011) that mediate some of the post-exercise glucose control improvements (Dube et al., 2011). These results of improving adiposity markers in HIIT and RT were displayed despite the higher energy expenditures per week from CT (90kcal·kg^−1^·min^−1^) than from HIIT and RT (~45kcal·kg^−1^·min^−1^ per session). However, it is well known that improvements in oxidative metabolism (i.e., decreases of carbohydrate oxidation and increases in fat oxidation during exercise) after ~10min of HIIT can be similar to ~40–60min of MICT (Burgomaster et al., 2008).

Concerning predict the residual effects of CT at ∆FPG_72h_ and ∆FI_72h_ by anthropometric/body composition or adiposity, stronger associations were found for body composition/anthropometry outcomes (model 4) with ∆FPG_72h_ (Table 2) and with ∆FI_72h_ (model 5; Table 3). Thus, as ∆FPG_72h_ was increased in the CT group with a larger effect size (+6.6mg/dl, *η*^2^: 0.76, Figure 3G), and the ∆FI_72h_ showed no real change, we presume that the residual effects for FPG and FI after 72-h post-training cessation are more related to body composition outcomes (i.e., decreases at adiposity outcomes) than increases at SMM that was also shown in both RT and CT groups (Figure 4A). Additionally, the ∆FPG_72h_ was significantly different to CG, as well as HIIT, and RT, but only the CT results showed a larger effect size, revealing the magnitude of difference to loss the residual effects from HIIT or RT alone, than by CT. As expected, the RT group showed significantly improved anthropometric parameters, whereas a significant loss of adipose tissue was mainly observed in the HIIT group. Accordingly, our data reveal that the metabolic adaptations after CT then can be explained by RT and HIIT, independently. However, considering that there may be an interference effect promoted by RT and HIIT, future studies of the cellular and molecular mechanisms might help to explain in part the mechanisms underlying the residual effects of RT and HIIT exercises on CT adaptations.

Our study presents some strengths that are important to highlight, such as (i) we used FPG, FI, and HOMA-IR indices, which are frequently used in the public health setting; (ii) we report RT and HIIT adaptations related to anthropometric/body composition outcomes regularly used in exercise training interventions; and (iii) the residual effects after CT training were not sufficient to improve the metabolic outcome in insulin-resistant patients. Our study is not without its limitations. For example, there was a lack of dietary control during the intervention, especially during the 24h to 72h in which metabolic outcomes were studied at pre- and post-test; however, the participants were reminded to maintain their current lifestyle habits. During the CT intervention, we first applied the RT component and then the HIIT exercises, and it is known that there is a potential interaction in the effects of exercise according to their order in the session. The sessions per week were different (HIIT 3, RT 2, and CT 3s/wk); however, these were displayed following international exercise recommendations per week (Bull et al., 2020). Also, the recovery period was different between HIIT and RT; however, the cardiorespiratory demands are higher in HIIT, with a need to re-establish the muscle and cardiorespiratory capacity using a double recovery period time. We also acknowledge that there are issues with the study design related to women’s reproductive health since we did not control for the menstrual cycle phase when performing pre-post testing, nor did we stratify groups by menopausal status. Also, assuming that participants maintained their energy intake patterns during the 72-h post-testing retention period, the CT group would have nearly twice as much energy excess compared to the RT or HIIT groups since the CT group expended more energy in the exercise sessions. This excess energy intake may explain part of the worsened FPG results in the CT group. Future studies could randomly assign the order of the intervention to avoid bias, include more sample size, to increase the control of the energy expenditure similarly at pre- and post-exercise cessation conditions, and to apply more than FPG the oral glucose tolerance test in which peripheral tissues key for glucose control as skeletal muscle mass is key more than FPG in which is the liver this role. Lastly, we did not perform any cellular and molecular analysis, which would improve the interpretation of our data and reveal potential mechanisms for more explanations.

## Conclusion

In summary, our results reveal that in insulin-resistant women HIIT decreases FPG, RT decreases FI 24-h post-training, and both types of exercise interventions when performed independently have a noteworthy residual effect on FPG and FI (post-72h) when compared with the CT intervention. However, it would be necessary to increase the residual effect study with larger sample sizes in clinical cohorts.

## Data Availability Statement

The original contributions presented in the study are included in the article/supplementary material, and further inquiries can be directed to the corresponding author.

## Ethics Statement

The studies involving human participants were reviewed and approved by the Family Healthcare Center Tomas Rojas Vergara (TRV) of Los Lagos, Chile (no. 0204015). The patients/participants provided their written informed consent to participate in this study. Written informed consent was obtained from the individual(s) for the publication of any potentially identifiable images or data included in this article.

## Author Contributions

CA, EC, GG, DA, and MI: conceptualization and visualization. CA, EC, GG, DA, MM-A, PD-F, and MI: methodology. CA, DA, MM-A, PD-F, and MI: software. CA, EC, MM-A, AA-M, and MI: validation. CA, EC, GG, DA, MM-A, and MI: formal analysis. CA, EC, GG, DA, MM-A, PD-F, FF, AA-M, and MI: investigation and writing—review and editing. CA: data curation and project administration. CA, EC, GG, and MI: writing—original draft preparation and supervision. CA, EC, and MI: funding acquisition. All authors have read and agreed to the published version of the manuscript.

## Funding

CA was funded partially by privates. MI was funded by a research grant PI17/01814 of the Ministerio de Economía, Industria y Competitividad (ISCIII, FEDER). EGC was funded by Conselho Nacional de Desenvolvimento Científico e Tecnológico (CNPq #303399/2018-0) during this project.

## Conflict of Interest

The authors declare that the research was conducted in the absence of any commercial or financial relationships that could be construed as a potential conflict of interest.

## Publisher’s Note

All claims expressed in this article are solely those of the authors and do not necessarily represent those of their affiliated organizations, or those of the publisher, the editors and the reviewers. Any product that may be evaluated in this article, or claim that may be made by its manufacturer, is not guaranteed or endorsed by the publisher.
